# Co-Application of Seaweed Extract (*Solieria filiformis*) and Silicon: Effect on Sporulation, Mycorrhizal Colonization, and Initial Growth of *Mimosa caesalpiniaefolia*

**DOI:** 10.3390/microorganisms13071581

**Published:** 2025-07-04

**Authors:** Isaac Alves da Silva, José Lucas Sousa de Andrade, Francisco Luan Almeida Barbosa, Murilo de Sousa Almeida, Marjory Lima Holanda Araújo, Adijailton Jose de Souza, Ademir Sergio Ferreira Araujo, Arthur Prudêncio de Araujo Pereira, Kaio Gráculo Vieira Garcia

**Affiliations:** 1 Biochemistry and Molecular Biology Department, Federal University of Ceará, Av. Mister Hull, 2977, Fortaleza 60440-900, Brazil; isaacalvesbiotec@gmail.com (I.A.d.S.); marjory.holanda@ufc.br (M.L.H.A.); 2 Soil Science Department, Federal University of Ceará, Av. Mister Hull, 2977, Fortaleza 60021-970, Brazil; lucasphy2@gmail.com (J.L.S.d.A.); luan02101994@gmail.com (F.L.A.B.); sousamuriloalmeida@gmail.com (M.d.S.A.); kaiovieira@ufc.br (K.G.V.G.); 3 Luiz de Queiroz College of Agriculture (ESALQ), University of São Paulo (USP), Piracicaba 13418-900, Brazil; adijailtonjsouza@alumni.usp.br; 4 Agricultural Science Center, Federal University of Piauí, Teresina 64049-550, Brazil; ademir@ufpi.edu.br

**Keywords:** plant–soil interactions, arbuscular mycorrhizae, biostimulants, silicate, sustainable practices

## Abstract

Seaweed extracts (SEs) and silicon (Si) are known to enhance plant growth under adverse conditions. However, their combined effects on arbuscular mycorrhizal fungi (AMF) are not yet fully understood. This study evaluated the effect of the co-application of an SE and Si on the AMF spore abundance, mycorrhizal colonization, and early growth of *Mimosa caesalpiniaefolia*. Plants were grown in a greenhouse for 70 days in soil with or without an SE (*Solieria filiformis*) and three Si levels (0, 150, and 300 mg kg^−1^). Growth parameters, AMF spore abundance, mycorrhizal colonization, and plant/soil chemical composition were assessed. SE and Si increased the plant height, stem diameter, number of leaves, and shoot dry mass, while higher Si levels reduced the root dry mass and length. Mycorrhizal colonization was highest (64%) at 150 mg kg^−1^ Si with SE, whereas AMF spore abundance decreased as Si increased. SE and 300 mg kg^−1^ Si raised the Si levels in the shoot, while root Si increased only at 300 mg kg^−1^ Si. Shoot Na increased at 300 mg kg^−1^ Si without SE, whereas K was highest at 150 mg kg^−1^ Si with SE. The soil pH, electrical conductivity, and Na increased at 300 mg kg^−1^ Si, while K and P decreased at this level without SE. These findings indicate that SE and Si co-application benefits early growth and may modulate mycorrhizal symbiosis, highlighting the importance of proper management to maximize plant and soil benefits.

## 1. Introduction

In agriculture, seaweed extracts (SEs) are widely used as biostimulants due to their complex composition, rich in polysaccharides such as laminarin, fucoidan, and alginate, which are predominant in brown macroalgae and extensively utilized in the formulation of these commercial extracts [1]. However, red macroalgae remain understudied for this purpose, with few reports in the literature regarding their biostimulant potential [2,3,4]. The application of an SE, either to the soil or via foliar spraying, can increase chlorophyll content, optimize photosynthesis and nutrient uptake, and improve water retention, thus promoting the growth of various agricultural crops and ecologically important plant species [5].

The red macroalga *Solieria filiformis* presents a promising biochemical composition for use as a biostimulant, containing 65.8% (*w*/*w*) carbohydrates, 9.6% (*w*/*w*) proteins, 1.7% (*w*/*w*) lipids, 7.0% (*w*/*w*) moisture, and 15.9% (*w*/*w*) ash, in addition to being rich in essential minerals such as calcium (Ca), potassium (K), magnesium (Mg), and phosphorus (P), as well as micronutrients like boron (B), copper (Cu), iron (Fe), manganese (Mn), and zinc (Zn) [6,7]. Evidence suggests that SEs may also positively influence soil microbiota by stimulating beneficial microorganisms, such as arbuscular mycorrhizal fungi (AMF) [8,9].

AMF are known to form mutualistic relationships with nearly 90% of land plants [10], playing a crucial role in promoting the absorption of water and essential nutrients such as P and nitrogen (N), as well as other beneficial elements like silicon (Si). Moreover, these interactions improve the plant’s ability to cope with both biotic and abiotic challenges [11,12,13], making AMF a valuable tool for sustainable agriculture. This is particularly relevant in degraded soils, which often exhibit low fertility and water scarcity. However, the effectiveness of mycorrhizal colonization depends on a complex signaling process between plant roots and AMF. In this context, flavonoids play an essential role as signaling molecules, activating spore germination and guiding hyphal growth toward roots [14].

Recently, various flavonoids have been identified in SEs, including hispidulin, as well as derivatives of gallocatechin and acacetin, which may stimulate mycorrhizal symbiosis [9]. Studies have demonstrated that SEs can promote AMF spore germination, hyphal growth, and accelerate mycorrhizal colonization. In *Medicago truncatula*, for instance, foliar application or immersion in SE resulted in the significantly higher expression of genes associated with the establishment of mycorrhizal symbiosis compared to controls [9].

Complementary to these effects, Si—an element widely distributed in the Earth’s crust—has been recognized for its role in mitigating environmental stresses and enhancing plant performance within the soil–plant–environment system [15,16]. Although Si is not classified as an essential element, its application can strengthen plant resistance against drought, salinity, and heavy metal toxicity [17,18]. Studies indicate that Si supplementation improves physiological processes and stimulates plant growth, particularly under adverse conditions [19,20,21]. Moreover, Si appears to strengthen mycorrhizal relationships by boosting photosynthetic activity in plants, supplying extra carbon to the fungi and curtailing lignin production [21].

However, the interaction between Si and AMF may vary depending on plant species and environmental conditions. While some plants with high Si accumulation are not significantly affected by AMF presence, in other species, such as strawberry, Si application favors mycorrhizal colonization and stimulates fungal structure formation, as well as increases Si uptake after mycorrhization [22]. In another study, with *Leucaena leucocephala*, Si application also increased mycorrhizal colonization; however, AMF spore abundance in the soil decreased with increasing Si levels [23]. Thus, it remains unclear how Si regulates mycorrhizal symbiosis and plant growth, especially in species adapted to nutrient-poor and semi-arid soils, such as *Mimosa caesalpiniaefolia*.

*M. caesalpiniaefolia* is a fast-growing, perennial leguminous species native to the Caatinga biome, widely recognized for its ecological importance and functional traits. It is capable of establishing symbiotic associations with both AMF [24] and nitrogen-fixing bacteria known as rhizobia [25], which makes it particularly suitable for studies on the interactions between biostimulants, soil microbiota, and plant nutrition. Its high adaptability to the edaphoclimatic conditions of the Caatinga [26], along with its frequent use in the recovery of degraded areas and agroforestry systems [27], reinforces its potential as a model species in sustainable land management strategies aimed at promoting soil health and resilience in semi-arid environments [28]. Beyond its ecological importance, *M. caesalpiniaefolia* exhibits significant agronomic value, serving multiple purposes such as forage for livestock, timber production, and green manure.

Although several studies have investigated the isolated effects of SEs and Si on plant growth, knowledge gaps remain regarding how the combined application of these inputs may influence mycorrhizal attributes and promote the development of plant species that have not been previously evaluated under this strategy. Thus, this study aims to evaluate the effect of the co-application of an SE (*S. filiformis*) and Si on mycorrhizal colonization, AMF sporulation, and the early growth of *M. caesalpiniaefolia*. We hypothesized that the combination of an SE (*S. filiformis*) and Si will promote increased mycorrhizal colonization, which in turn will stimulate AMF spore abundance in the soil, ultimately enhancing the growth of this species.

## 2. Materials and Methods

### 2.1. Study Area and Experimental Soil

The study was conducted in a greenhouse at the Department of Soil Science, Federal University of Ceará (UFC), located at the Pici Campus in Fortaleza, Ceará, Brazil (3°45′47″ S; 38°31′23″ W; 47 m altitude). According to the Köppen–Geiger classification, the region has a tropical climate with a dry winter (Aw), an average annual rainfall of 1600 mm, and a mean temperature of 27 °C [29]. Soil samples were collected from a depth of 0–20 cm in a native forest area within the Urban Agriculture Teaching and Research Center (NEPAU) at the UFC. The samples were passed through a 2 mm mesh sieve to remove coarse particles, homogenized, and stored in plastic bags. The chemical characterization of the soil was performed at the Soil, Water, and Plant Laboratory of the UFC following the methodology described by [30] (Table 1).

### 2.2. Seaweed Type and Production Process

The seaweed used in the extract was *S. filiformis*, a red algae species commonly found along the Brazilian coast. The *S. filiformis* used in this study was harvested from cultivation structures known as colonized modules, maintained at sea by the Flecheiras and Guajiru Seaweed Producers Association (APFG), in collaboration with the Algae Biotechnology and Bioprocess Laboratory (BioAP) of the Department of Biochemistry and Molecular Biology at the UFC. The collection site was on the western coast of Ceará, in the municipality of Trairi, at Flecheiras Beach, Brazil. After collection, the algae were stored frozen in their natural state.

To produce the extract, 500 g of *S. filiformis* was ground in an electric mill with 2.5 L of distilled water for 1 min and 30 s. The mixture was then heated on a hot plate (MYLABOR-model AG-10 [https://www.lojanetlab.com.br, accessed 2 July 2025]) until it reached 80 °C and maintained at this temperature for 1 h under constant mechanical stirring at 140 RPM. As some water evaporated during heating, it was replenished to maintain the initial volume. The solution was subsequently filtered through a fine-mesh nylon fabric at 80 °C to prevent gelatinization, which could hinder the filtration process, given that gelation begins at 45 °C. The final filtrate was stored in a refrigerator for later application to the plants. A detailed characterization of the chemical composition of *S. filiformis* used in this study is available in [7].

### 2.3. Process and Experimental Design

The experiment followed a completely randomized design (CRD) in a 2 × 3 factorial arrangement, considering (i) the presence or absence of *S. filiformis* seaweed extract (SE) in the soil and (ii) three levels of silicon (0, 150, and 300 mg kg^−1^). The study included 5 replicates per treatment, totaling 30 experimental units.

The soil was placed into 1 L pots, each containing 1 kg of substrate. To minimize drainage and nutrient leaching, the pots were lined with plastic bags. We chose to use non-sterilized soil in this experiment in order to maintain edaphic conditions as close as possible to those found in the natural environment. This approach aims to allow more representative microbial interactions, resulting in data with greater ecological relevance. Si was incorporated at three levels, 0, 150, and 300 mg kg^−1^, using sodium silicate (Na_2_SiO_3_) as the source. The Si levels were selected based on previous studies by [23,31]. Following Si treatment, the soil underwent thorough homogenization and was incubated for ten days. The seaweed extract, supplied by the Algae Biotechnology and Bioprocess Laboratory at the UFC, was applied to the soil at a 10% (*v*/*v*) concentration. This concentration was selected based on prior studies [32,33] that demonstrated its effectiveness. Before sowing, *M. caesalpiniaefolia* seeds were exposed to 70% ethanol for 30 s to reduce surface tension, then disinfected using a 1% sodium hypochlorite solution for 10 min. Subsequently, the seeds were rinsed with sterile distilled water to eliminate any remaining hypochlorite residue [34]. Three seeds were planted per pot, and 10 days after seedling emergence, thinning was carried out to retain a single plant per pot. The soil was irrigated daily to maintain approximately 60% of field capacity. Field capacity was estimated based on the concept of total available water (TAW) described by [35], considering the soil bulk density, effective root zone depth, and typical water content values at field capacity and permanent wilting point for the soil type. Water replacement was performed daily using pot lysimeters to compensate for evapotranspiration losses. The experiment lasted for 70 days after sowing (DASs).

### 2.4. Plant Growth Parameters

At 70 DASs, the plants were collected, placed in paper bags, and dried in a forced-air oven (PROLAB-model SSDicr-150 [https://www.prolab.com.br, accessed 2 July 2025]) at 65 °C for three days to determine the shoot dry mass (SDM). The roots were rinsed with running water and dried following the same procedure to obtain the root dry mass (RDM). The plant height (H) was measured with a graduated ruler from the soil surface to the plant apex and expressed in centimeters. The root length (RL) was also measured with a graduated ruler from the soil insertion point to the root tip. The stem diameter (SD) was recorded 5 cm above the soil surface using a digital caliper (MTX-model 316119 [https://mtxtools.ru, accessed 2 July 2025]) and expressed in millimeters. The number of leaves (NL) was determined through direct counting and expressed as the NL per plant.

### 2.5. Quantification of Mycorrhizal Colonization and Spore Abundance

Mycorrhizal colonization (MC) was assessed by clearing the roots with a 10% potassium hydroxide (KOH) solution, as outlined by [36]. The samples were placed in open tubes containing a 10% KOH solution and subjected to a preheated water bath (MYLABOR-model SSDc-10 [https://www.lojanetlab.com.br, accessed 2 July 2025]) maintained at 80 °C for 1 h, with the solution being replaced every 20 min. After this period, the tubes were removed from the water bath, the KOH solution was discarded, and a 3% hydrogen peroxide (H_2_O_2_) solution was added. Subsequently, the roots were washed with distilled water. Following the clearing process, the roots were stained by adding a 5% acidified blue ink solution (Parker Quink [https://www.parkerpen.com, accessed 2 July 2025]) to the tubes, which were again placed in the water bath at 80 °C for 10 min, following the method by [37]. After staining, the samples were removed, washed with a 5% acetic acid solution, and stored in a preservative solution composed of equal volumes of glycerol, lactic acid, and distilled water (1:1:1 *v*/*v*). For microscopic analysis, slides were prepared with ten root fragments, each approximately 1 cm long, and examined using an optical microscope (BIOFOCUS-model Blue-1600 [https://www.ionlab.com.br, accessed 2 July 2025]). Mycorrhizal colonization (%) was assessed following the methodology described by [38].

The abundance of AMF spores in the soil (AS) was assessed using the wet sieving method, following the protocol described by [39]. For this, 100 g of soil from each sample was mixed with ~500 mL of water and blended at a high speed for 15 s in a Mondial Blender. The resulting suspension was then poured through a series of sieves with mesh sizes of 106 and 44 µm. All particles retained on the 44 µm sieve were collected and centrifuged with a 70% sucrose solution at 3500 rpm for 5 min. The AMF spores in suspension were filtered (44 µm mesh), rinsed with water, transferred to Petri dishes, and quantified by direct counting under a stereomicroscope (OLEN-model TECNIVAL-SQF-F [https://www.lojanetlab.com.br, accessed 2 July 2025]).

### 2.6. Silicon in Shoots and Roots and Phosphorus, Sodium, and Potassium in Shoots

The Si content in the roots and shoots of *M. caesalpiniaefolia* was extracted using 30% hydrogen peroxide and 50% sodium hydroxide. The samples were then placed in a water bath (MYLABOR-model SSDc-10 [https://www.lojanetlab.com.br, accessed 2 July 2025]) at 85 °C for 1 h until complete gas release occurred. Next, they underwent digestion in a semi-open Falcon tube using an autoclave (PHOENIX-model AV-75 [https://phoenix.ind.br, accessed 2 July 2025]) at 123 °C and 1.5 atm pressure for 1 h [40]. Si determination in plant tissues was performed through colorimetry at 410 nm, following the procedures described by [40]. The concentrations of P, sodium (Na), and K in the shoots of *M. caesalpiniaefolia* were extracted using 1 mol L^−1^ HCl [30]. P was determined by colorimetry (KASVI-model K37-VIS [https://kasvi.com.br, accessed 2 July 2025]) at a wavelength of 660 nm, while Na and K were analyzed using flame photometry (DIGIMED-model DM-62 [https://www.digimed.ind.br, accessed 2 July 2025]) [30].

### 2.7. Soil Chemical Analysis

Electrical conductivity (EC) and soil solution pH were measured in water (1:2.5 soil-to-distilled water ratio). The pH was determined using a potentiometer (R-TEC-7/2-MP Tecnal [https://tecnal.com.br, accessed 2 July 2025]), while EC was measured with a conductivity meter (DDS-11C MFC:2409087 Meter [https://impac.com.br, accessed 2 July 2025]) [30]. Soil Si content was extracted using a 0.5 M acetic acid solution and quantified by colorimetry (KASVI-model K37-VIS [https://kasvi.com.br, accessed 2 July 2025]) at a wavelength of 660 nm, following the procedures described by [41]. Soil P content was extracted using Mehlich 1 solution (0.05 mol L^−1^ HCl and 0.0125 mol L^−1^ H_2_SO_4_) and determined by colorimetry (KASVI-model K37-VIS [https://kasvi.com.br, accessed 2 July 2025]) at a wavelength of 660 nm. Na and K were extracted using 1N ammonium acetate and determined by flame photometry (DIGIMED-model DM-62 [https://www.digimed.ind.br, accessed 2 July 2025]) [30].

### 2.8. Statistical Analysis

Statistical analyses were conducted to assess the data distribution and variance homogeneity. Normality was evaluated using the Shapiro–Wilk test, and Levene’s test was applied to verify the homogeneity of variances. When the data met the normality assumption and exhibited homogeneous variances, a two-way analysis of variance (ANOVA) was performed using the F test (*p* ≤ 0.05). When significant differences were detected, mean comparisons were conducted using the Scott–Knott test (*p* ≤ 0.05). All statistical analyses were performed using the AgroEstat software (version 1.1.0.712).

## 3. Results

### 3.1. Plant Growth

**Figure 1:**
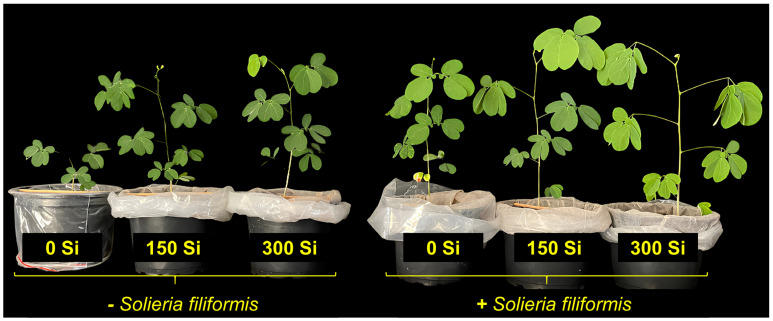
Growth of *M. caesalpiniaefolia* plants in the absence (−*S. filiformis*) and presence (+*S. filiformis*) of *S. filiformis* seaweed extract, under three levels of Si applied to the soil (0 mg kg^−1^, 150 mg kg^−1^, and 300 mg kg^−1^).

**Figure 2:**
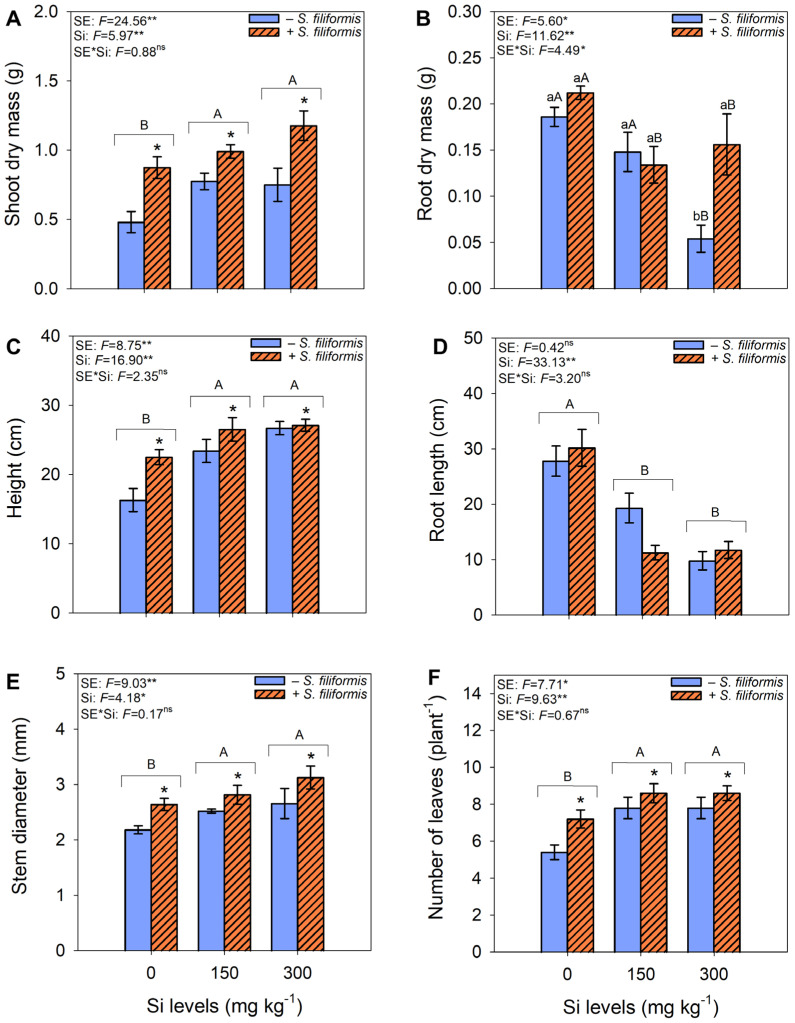
Growth parameters of *M. caesalpiniaefolia* under different treatments. Shoot dry mass (**A**), root dry mass (**B**), plant height (**C**), root length (**D**), stem diameter (**E**), and leaf number (**F**). The values represent the mean of five replicates ± standard error. Uppercase letters indicate significant differences among Si levels within the same seaweed extract treatment (i.e., −*S. filiformis* or +*S. filiformis*). Uppercase letters placed above brackets, when present, indicate significant differences among Si levels, regardless of seaweed extract application. Lowercase letters and asterisks indicate significant differences between plants with and without seaweed extract (i.e., −*S. filiformis* or +*S. filiformis*) within the same Si level, according to the Scott–Knott test (*p* ≤ 0.05). *, **, ns: *F*-test significance levels at *p* ≤ 0.05, *p* ≤ 0.01, and not significant, respectively.

*Plants of M. caesalpinifolia* Benth. exhibited improved growth with the application of *S. filiformis* seaweed extract and Si (Figure 1). The application of *S. filiformis* seaweed extract increased the shoot dry mass production, plant height, stem diameter, and leaf number, regardless of Si levels. Additionally, at Si levels of 150 and 300 mg kg^−1^, these variables increased regardless of the application of *S. filiformis* seaweed extract (Figure 2A,C,E,F). On the other hand, the absence of Si application resulted in higher root dry mass production and root length (Figure 2B,D).

### 3.2. Abundance of AMF Spores in the Soil and Mycorrhizal Colonization

**Figure 3:**
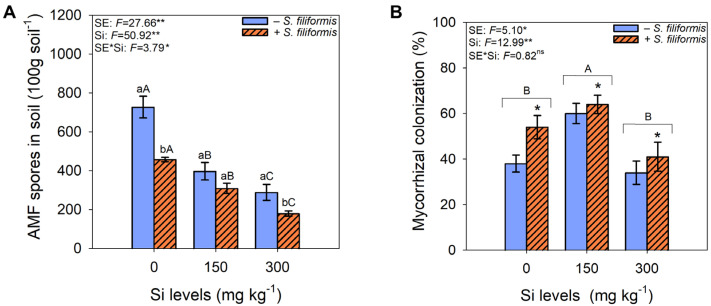
Abundance of AMF spores in the soil (**A**) and total mycorrhizal colonization (**B**) in *M. caesalpiniaefolia* plants subjected to different treatments. The values in the figure represent the mean of five replicates ± standard error. Uppercase letters indicate significant differences among Si levels within the same seaweed extract treatment (i.e., −*S. filiformis* or +*S. filiformis*). Uppercase letters placed above brackets, when present, indicate significant differences among Si levels, regardless of seaweed extract application. Lowercase letters and asterisks indicate significant differences between plants with and without seaweed extract (i.e., −*S. filiformis* or +*S. filiformis*) within the same Si level, according to the Scott–Knott test (*p* ≤ 0.05). *, **, ns: *F*-test significance levels at *p* ≤ 0.05, *p* ≤ 0.01, and not significant, respectively.

The abundance of AMF spores in the soil significantly decreased with increasing Si levels, particularly at 300 mg kg^−1^ of Si, and with the application of *S. filiformis* seaweed extract (Figure 3A). The highest percentages of mycorrhizal colonization were observed at 150 mg kg^−1^ of Si, regardless of *S. filiformis* seaweed extract application, and in the presence of *S. filiformis* seaweed extract, regardless of Si levels (Figure 3B).

**Figure 4:**
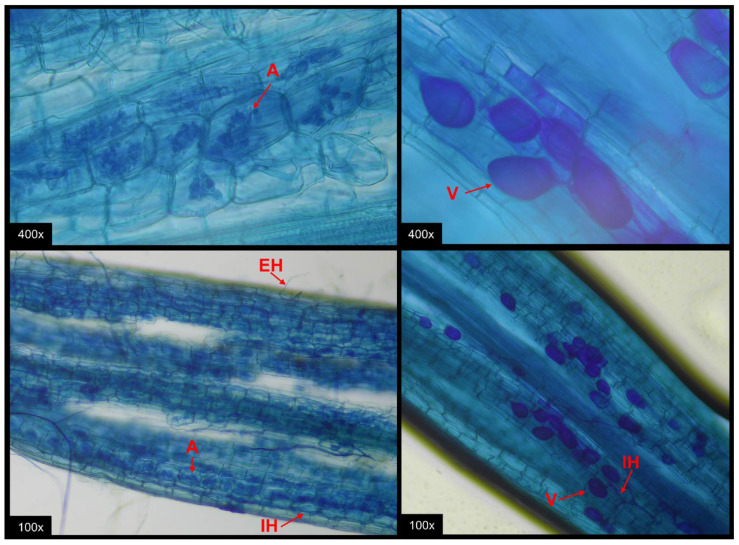
Optical microscope photograph of *M. caesalpinieafolia* roots after staining. V: vesicle; IH: intraradicular hypha; EH: extraradicular hypha; A: arbuscule.

The roots of *M. caesalpiniaefolia* colonized by AMF exhibited endogenous structures, including Arum-type arbuscules, vesicles, and intraradical and extraradical hyphae (Figure 4).

### 3.3. Silicon in Shoots and Roots and Phosphorus, Sodium, and Potassium in Shoots

**Figure 5:**
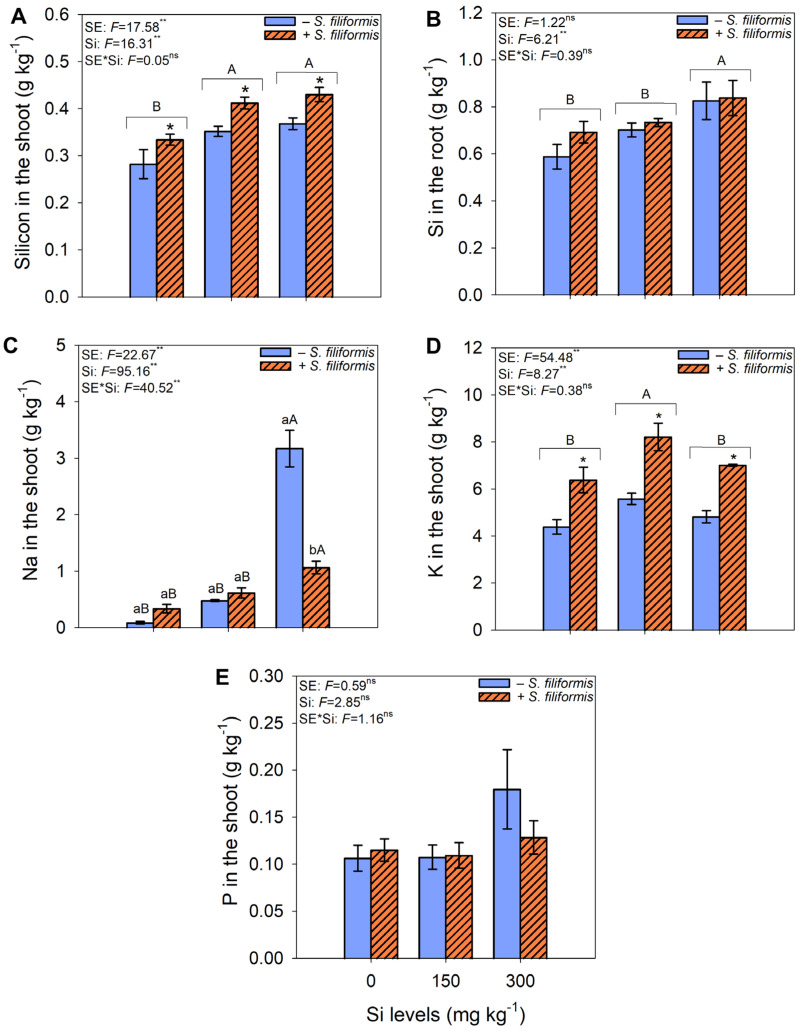
Silicon in shoots (**A**) and roots (**B**) and Na (**C**), K (**D**), and P (**E**) in shoots in *M. caesalpiniaefolia* plants subjected to different treatments. The values in the figure represent the mean of five replicates ± standard error. Uppercase letters indicate significant differences among Si levels within the same seaweed extract treatment (i.e., −*S. filiformis* or +*S. filiformis*). Uppercase letters placed above brackets, when present, indicate significant differences among Si levels, regardless of seaweed extract application. Lowercase letters and asterisks indicate significant differences between plants with and without seaweed extract (i.e., −*S. filiformis* or +*S. filiformis*) within the same Si level, according to the Scott–Knott test (*p* ≤ 0.05). *, **, ns: *F*-test significance levels at *p* ≤ 0.05, *p* ≤ 0.01, and not significant, respectively.

The highest Si concentrations in the shoot were observed at Si levels of 150 and 300 mg kg^−1^ in the soil, regardless of *S. filiformis* seaweed extract application, and in the presence of *S. filiformis* seaweed extract, regardless of the applied Si levels (Figure 5A). The highest Si concentrations in the root were found at 300 mg kg^−1^ of Si applied to the soil (Figure 5B). The highest Na concentrations in the shoot were observed at 300 mg kg^−1^ of Si, in the absence of *S. filiformis* seaweed extract (Figure 5C). K concentrations were higher at 150 mg kg^−1^ of Si, regardless of *S. filiformis* seaweed extract application, and with the application of *S. filiformis* seaweed extract, regardless of the Si levels applied to the soil (Figure 5D). There was no significant effect on P concentrations in the shoot in response to the analyzed treatments (Figure 5E).

### 3.4. Soil Chemical Analysis

The application of *S. filiformis* seaweed extract did not significantly affect the soil pH, EC, or Na content (Table 2). An increase in the soil solution pH was observed at the highest Si dose (300 mg kg^−1^), while EC rose with both 150 and 300 mg kg^−1^ of Si (Table 2). The greatest availability of Si in the soil occurred at 300 mg kg^−1^ of Si, specifically in the absence of the seaweed extract (Table 2). Likewise, the highest Na content was recorded at this Si rate. K concentrations were elevated at 0 mg kg^−1^ of Si without the extract and at 150 mg kg^−1^ with the extract (Table 2). No significant differences were observed in P content across treatments, with the exception of the application of 300 mg kg^−1^ of Si in the presence of *S. filiformis* SE (Table 2).

## 4. Discussion

The application of a seaweed extract (*S. filiformis*) and Si maximized the early growth of *Mimosa caesalpiniaefolia*, possibly due to the bioactive composition of the seaweed extract. This extract contains polysaccharides, phytohormones, lipids such as fatty acids and sterols, pigments like carotenoids, oxylipins, minerals, peptides, amino acids, and proteins, compounds that promote plant growth [5]. Furthermore, there is evidence that seaweed extracts stimulate AMF, which support plant development [8,9], an effect also observed in this study. However, it is worth noting that the seaweed extract used in our study was subjected to heating and filtration, processes that may have altered its initial composition. Some compounds may have been retained in the discarded residue; however, the gelled extract retains sulfated polysaccharides from the seaweed cell wall, capable of interacting with soil minerals and promoting their gradual release. This mechanism reduces leaching and improves nutrient availability for plants, favoring their development [6,42].

The gel formulation of the seaweed extract used in our study reduces dispersion by rain and leaching, prolonging root contact and improving nutrient absorption. This factor is particularly relevant for seedlings intended for reforestation, which need to develop autonomy in the final environment, where input availability is limited. The interaction between Si and seaweed extracts may provide better conditions for plant growth since both inputs enhance water and nutrient absorption while strengthening plant cell structure [43]. Si contributes to silica deposition in cell walls, increasing mechanical resistance and reducing water stress, while bioactive seaweed compounds promote beneficial microbial activity and optimize plant metabolism [44]. The absence of Si resulted in greater dry mass production and root length. This effect may be related to the fact that, under certain conditions, Si reduces root growth without compromising the shoot [45]. Reduced root expansion may indicate greater efficiency in resource uptake since Si contributes to water use efficiency and structural resistance in the shoot [46].

The abundance of AMF spores in the soil decreased with the application of the seaweed extract and increasing levels of Si. This behavior may be related to the lower root density observed under these conditions and the signaling dynamics between plants and AMF in the rhizosphere. A study demonstrated that plants under greater environmental stress release root exudates, such as strigolactones and flavonoids, to stimulate the establishment of symbiosis with AMF [47]. These molecules function primarily as exo-signals that promote hyphal branching and facilitate root colonization. In our study, however, even with improved plant nutritional status and reduced stress due to SE and Si application, AMF colonization increased. This suggests that the mechanisms driving colonization in our experimental conditions may not be directly dependent on the elevated exudation of signaling compounds. On the other hand, a reduction in AMF spore abundance in the soil was observed. Since strigolactones and flavonoids are not directly involved in sporulation, this reduction may instead be related to changes in carbon allocation by the host plant, shifts in AMF life strategies, or even the suppression of sporulation under more favorable root-colonizing conditions. Similar findings were reported by [23], highlighting that increased colonization does not necessarily correlate with higher spore production.

Mycorrhizal colonization in *M. caesalpiniaefolia* increased in response to the application of the seaweed extract and Si. Although this treatment led to reduced AMF sporulation, it did not negatively affect the plant. On the contrary, greater mycorrhizal colonization resulted in significant benefits for *M. caesalpiniaefolia* growth, indicating that spore abundance and mycorrhizal colonization are not always directly correlated. Studies suggest that seaweed extract application stimulates mycorrhization compared to its absence [8]. This effect may be related to the increased expression of genes associated with mycorrhizal symbiosis establishment, such as those involved in infectivity and colonization efficiency, as observed in *Medicago truncatula* [9]. Additionally, Si may favor mycorrhizal colonization by modulating the availability of dissolved organic compounds in the soil, increasing the supply of carbon sources for AMF [21]. Furthermore, there is evidence that Si influences the metabolism of phenolic substances, including flavonoids, which play a crucial role in signaling and recruiting AMF to the host plant rhizosphere [48]. Thus, although Si deposition in root cell walls is traditionally associated with a potential barrier to fungal infection, the present study suggests that this element may, in fact, create a biochemically favorable environment for mycorrhizal colonization in *M. caesalpiniaefolia*. Therefore, our findings suggest that the interaction between the seaweed extract and Si fosters a more conducive environment for mycorrhizal symbiosis, promoting greater colonization regardless of reduced AMF sporulation in the soil.

It is important to acknowledge that, under natural soil conditions, AMF symbioses occur within complex networks formed by multiple fungal species interacting with diverse plant roots across the soil matrix. Such networks are essential for nutrient transfer, colonization dynamics, and spore production. However, our experimental design, based on pot conditions with a single plant species, does not replicate the full complexity of field scenarios. Consequently, although we quantified root colonization and spore abundance in the soil, we did not assess the development of the extraradical mycelial network nor identify the AMF species involved. This constitutes a limitation of the study, as the diversity and connectivity of AMF networks can influence both colonization and sporulation. Therefore, future studies under more complex or field-based conditions are necessary to expand the understanding of AMF network functioning and its implications for symbiotic performance and ecosystem services.

The application of seaweed extract influenced Si uptake in *M. caesalpiniaefolia*, resulting in the higher accumulation of this element in the shoot, while the Si levels in the roots increased proportionally with higher Si application to the soil. These results indicate that despite the reduction in root growth observed at high Si doses, the absorption and storage of this element in the root remained active, corroborating the findings of [45]. This effect may be related to the intensification of mycorrhizal colonization since AMF can enhance Si uptake by expanding the soil exploration surface through their hyphal network, acting as an extension of the root system [8,49]. Moreover, the composition of the seaweed extract may have contributed to increased Si levels in the plant shoots. Studies indicate that green and red macroalgae have higher Si concentrations compared to brown macroalgae [50]. Although we applied a highly soluble silicon source, it is important to recognize that in natural soils, silicate-solubilizing bacteria can also enhance Si availability by transforming insoluble minerals into forms absorbable by plants. These bacteria act through acidification and organic acid production, contributing to nutrient cycling and potentially interacting with AMF to improve plant nutrition and stress tolerance [20].

Thus, the interaction between the bioactive compounds of *S. filiformis* seaweed extract and soil Si availability may have favored the absorption and transport of the element, leading to its greater accumulation in shoot tissues. However, it is important to acknowledge that the speciation of Si in the soil solution was not determined in this study. Since sodium silicate is a highly soluble source, high application rates may have exceeded the solubility threshold of monomeric silicic acid, potentially leading to polycondensation and reduced bioavailability. This represents a limitation for the precise interpretation of Si dynamics under our experimental conditions.

The high Si concentration influenced sodium uptake by the plant in our study, possibly due to the use of sodium silicate as a Si source, which contains Na^+^ in its composition. Although Si is known to mitigate salt stress by regulating Na^+^ transport in plant tissues [45,51,52], the presence of Na^+^ in the fertilizer may have contributed to its accumulation in the shoot. However, the observed Na^+^ levels did not indicate toxicity for *M. caesalpiniaefolia* [53]. The lower Na^+^ translocation in the presence of the *S. filiformis* seaweed extract may be associated with its gelatinous matrix, rich in sulfated polysaccharides, which have negative charges capable of retaining cations in the soil.

The *S. filiformis* seaweed extract increased the K levels in the shoot of *M. caesalpiniaefolia*, likely due to the supply of this nutrient and plant hormones that stimulate growth, favoring its absorption [8,54]. Additionally, the symbiosis with AMF, enhanced by the seaweed extract and Si, expands the root absorption area, boosting K uptake [9]. Si application to the soil also contributes to K uptake by improving its availability and facilitating transport within the plant, potentially reducing K fixation in soil particles and increasing its mobility in solution [55]. Si further strengthens the cell structure, reducing K losses through exudation and regulating its redistribution in plant tissues [56]. Studies indicate that under K deficiency, Si can stimulate compensatory mechanisms, promoting plant growth and nutrient homeostasis [51]. In our study, there were no significant differences in the P content in the shoot of *M. caesalpiniaefolia* among the treatments. This result may be related to the experimental period, which was possibly insufficient for the *S. filiformis* extract to significantly release P into the soil, thus limiting its availability to plants. As reported by [57], the mineralization of organic P can vary with the incubation time, influencing the nutrient’s availability to plants.

The soil application of Si directly influenced the pH of the soil solution and electrical conductivity (EC). The increase in the pH, especially observed in the treatment with the highest level of Si, is likely due to the alkalinizing effect of sodium silicate. This effect occurs because silicate anions act as weak bases, neutralizing H^+^ ions in the soil solution. Additionally, Na^+^ can compete with H^+^ for exchange sites on soil colloids, displacing H^+^ into the solution and promoting its neutralization [51,58]. On the other hand, the increase in the EC can be attributed to the dissociation of Na_2_SiO_3_ in the soil, which releases Na^+^ ions and silicate anions (SiO_4_^4−^), increasing the concentration of dissolved ions in the soil solution and, consequently, the EC [52].

Soil Si availability increased with the application of increasing doses of this element, as expected. In contrast, the application of the *S. filiformis* seaweed extract resulted in a reduction in soil Si availability. This decrease may be related to the higher accumulation of Si in the shoot of *M. caesalpiniaefolia* under this treatment, indicating greater uptake of the element. This effect may be associated with the intensification of mycorrhizal colonization, as observed in our study, since arbuscular mycorrhizal fungi (AMF) are also known to enhance Si uptake by plants [49].

The increase in the soil sodium content with higher Si levels can be attributed to the chemical composition of common Si sources, which often include soluble salts like sodium or potassium silicate. Si application may also alter the soil’s colloidal structure and ion exchange processes, facilitating the release of Na^+^ into the soil solution [59]. SEs, however, may moderate this effect through ionic complexation by sulfated polysaccharides or by enhancing microbial activity that influences cation dynamics [60].

The soil K levels decreased with the application of Si, likely due to the increased uptake of this nutrient by plants under higher Si treatments, resulting in reduced K availability in the soil. Potassium availability is influenced by competition with other cations and by plant uptake during periods of rapid growth. Moreover, the *S. filiformis* SE can enhance K release from soil minerals through the action of organic compounds such as humic, fulvic, and phenolic acids, which aid in nutrient solubilization [60]. The SE may also stimulate the activity of rhizospheric microorganisms and AMF, which play key roles in the mobilization of K and P. Notably, the effects on P availability in the soil were more pronounced in the presence of the SE and under the highest Si application level. Phosphorus dynamics are particularly complex due to its high reactivity, but SEs can reduce P fixation by promoting the release of chelating substances and enhancing microbial activity.

## 5. Conclusions

The co-application of *S. filiformis* SE and Si promotes the early growth of *M. caesalpiniaefolia*, particularly at Si levels of 150 and 300 mg kg^−1^, enhancing its development. Si, in the form of Na_2_SiO_3_, and the *S. filiformis* SE reduce AMF spore abundance in the soil cultivated with *M. caesalpiniaefolia*, possibly due to lower root density and reduced exudate release. The application of *S. filiformis* SE and Si increased mycorrhizal colonization in *M. caesalpiniaefolia*, benefiting plant growth despite the reduction in AMF sporulation, suggesting that symbiosis can be stimulated independently of spore abundance. The co-application of *S. filiformis* SE and Si shows potential for promoting the growth of *M. caesalpiniaefolia* and modulating its interaction with AMF, partially confirming the hypothesis of this study. However, further field studies are needed, considering different soil types, plant species, and a longer experimental period to assess AMF sporulation dynamics, mycorrhizal colonization, and nutrient availability in the soil solution over time.

## Figures and Tables

**Table 1:** Chemical and physical characterization of the soil collected from the Urban Agriculture Teaching and Research Center (NEPAU) at the UFC, Fortaleza, CE, Brazil.

pH	EC	Mg^2+^	Na^+^	K^+^	H + Al	Al^3+^	BS	AS	ESP
(H_2_O)	(dS/m)	(cmol_c_/kg)					(%)	(%)	(%)
5.0	0.9	0.4	0.6	0.18	4.46	0.5	41	14	1
N	OM	P	Bulk Density	Coarse Sand	Fine Sand	Silt	Clay	Natural Clay	Textural Class
(g/kg)	(g/kg)	(mg/kg)	g/cm^3^	(g/kg)					-
1.36	21.39	37	1.6	586	282	95	37	4	Loamy Sand

Note: EC = electrical conductivity, BS = base saturation percentage, AS = aluminum saturation percentage, ESP = exchangeable sodium percentage, OM = organic matter.

**Table 2:** The pH, EC, and the contents of Si, Na, K, and P in the soil cultivated with *M. caesalpiniaefolia* under different treatments. Uppercase letters indicate significant differences among Si levels within the same seaweed extract treatment (i.e., −*S. filiformis* or +*S. filiformis*). Lowercase letters indicate significant differences between plants with and without seaweed extract (i.e., −*S. filiformis* or +*S. filiformis*) within the same Si level, according to the Scott–Knott test (*p* ≤ 0.05). *, **, ns: *F*-test significance levels at *p* ≤ 0.05, *p* ≤ 0.01, and not significant, respectively.

Treatments		pH	EC	Si	Na	K	P
SE	Si (mg kg^−1^)	(H_2_O)	(µS cm^−1^)	(mg kg^−1^)	(cmol_c_ kg^−1^)	(cmol_c_ kg^−1^)	(mg kg^−1^)
−*S. filiformis*	0	4.60 ± 0.12 C	204.60 ± 16.54 B	2.49 ± 0.08 aC	0.15 ± 0.003 C	0.04 ± 0.001 aA	20.95 ± 1.29 aA
	150	5.55 ± 0.07 B	227.92 ± 17.77 A	3.96 ± 0.19 aB	0.54 ± 0.008 B	0.03 ± 0.0009 bB	21.31 ± 1.12 aA
	300	6.21 ± 0.05 A	255.60 ± 7.89 A	7.87 ± 0.64 aA	0.87 ± 0.031 A	0.01 ± 0.001 bC	16.36 ± 0.79 bB
+*S. filiformis*	0	4.56 ± 0.02 C	210.56 ± 8.18 B	2.58 ± 0.17 aB	0.16 ± 0.003 C	0.03 ± 0.001 bA	20.95 ± 1.00 aA
	150	5.68 ± 0.07 B	263.00 ± 11.15 A	3.45 ± 0.13 aB	0.55 ± 0.005 B	0.04 ± 0.001 aA	22.67 ± 0.42 aA
	300	6.31 ± 0.08 A	240.20 ± 8.12 A	6.01 ± 0.17 bA	0.86 ± 0.011 A	0.03 ± 0.001 aB	21.48 ± 1.10 aA
*F* test							
SE		0.95 ns	0.72 ns	9.70 **	0.21 ns	20.04 **	7.00 *
Si		226.60 **	6.74 **	116.93 **	1213.11 **	63.10 **	4.86 *
SE*Si		0.65 ns	2.12 ns	5.56 *	0.45 ns	33.53 **	2.52 *

## Data Availability

The original contributions presented in this study are included in the article. Further inquiries can be directed to the corresponding author.
